# Two new genera and species of the Gigantometopini (Hemiptera, Heteroptera, Miridae, Isometopinae) from Borneo with remarks on the distribution of the tribe

**DOI:** 10.3897/zookeys.941.47432

**Published:** 2020-06-16

**Authors:** Artur Taszakowski, Junggon Kim, Claas Damken, Rodzay A. Wahab, Aleksander Herczek, Sunghoon Jung

**Affiliations:** 1 Institute of Biology, Biotechnology and Environmental Protection, Faculty of Natural Sciences, University of Silesia in Katowice, Bankowa 9, 40-007 Katowice, Poland; 2 Laboratory of Systematic Entomology, Department of Applied Biology, College of Agriculture and Life Sciences, Chungnam National University, Daejeon, South Korea; 3 Institute for Biodiversity and Environmental Research, Universiti Brunei Darussalam, Jalan Universiti, BE1410, Darussalam, Brunei; 4 Department of Smart Agriculture Systems, College of Agriculture and Life Sciences, Chungnam National University, Daejeon, South Korea

**Keywords:** Asia, biodiversity, distribution, jumping tree bugs, plant bugs, true bugs

## Abstract

Two new genera, each represented by a single new species, *Planicapitus luteus* Taszakowski, Kim & Herczek, **gen. et sp. nov.** and *Bruneimetopus simulans* Taszakowski, Kim & Herczek, **gen. et sp. nov.**, are described from Borneo. Detailed photographs of male habitus and genital structures are presented. The checklist with distributional records for all known taxa of Gigantometopini is also provided.

## Introduction

The Isometopinae are a highly autapomorphic group possessing paired ocelli which are absent in all other members of the plant bug family Miridae (Herczek 1993, Namyatova and Cassis 2016, Yasunaga et al. 2017). This subfamily was considered as the sister group to all other subfamilies based on morphology (Schuh 1974, 1976), but recent works using molecular data do not support this hypothesis (Schuh et al. 2009, Jung and Lee 2012). Therefore, additional works are needed to understand phylogenetic position of this subfamily.

The group has a worldwide distribution, but the majority of known taxa are thermophilic, and occur in the tropics, subtropics, and warm temperate climate zones (Eyles 1971, Schuh 2002–2013, Cassis and Schuh 2012, Namyatova and Cassis 2016, Yasunaga et al. 2017). Due to scarce information on habits, biology, and food preference, the representatives are relatively rare in collections with many species known from singletons or only a handful of specimens (Eyles 1971, Namyatova and Cassis 2016, Herczek et al. 2018). Currently, six tribes, 45 genera and more than 250 species of Isometopinae are known (Namyatova and Cassis 2016, Yasunaga et al. 2016, 2017, Krüger 2018, Herczek et al. 2018) of which 19 species are fossil taxa (Herczek 1993, Schuh 2002–2013, Herczek and Popov 2012, 2014). The Isometopini and Myiommini are the most species-rich isometopine tribes known worldwide (Namyatova and Cassis 2016). The Electromyiommini is an extinct tribe and contains four genera and 14 species; all were described from Baltic amber (Herczek 1993). The Diphlebini includes only a single genus, *Diphleps* Bergroth, 1924 (Schuh 2002–2013). Yasunaga et al. (2017) created the new tribe Sophianini comprising two genera previously classified within Myiommini: *Alcecoris* McAtee & Malloch, 1924 and *Sophianus* Distant, 1904. Sophianini includes ten species (Yasunaga et al. 2017).

The last tribe is Gigantometopini created by Herczek (1993) to accommodate a single species *Gigantometopus rossi* Schwartz & Schuh, 1990. In 2002 *Isometopidea gryllocephala* Miyamoto, Yasunaga & Hayashi, 1996 was transferred to a newly created genus *Astroscopometopus* Yasunaga & Hayashi and its inclusion to the tribe Gigantometopini was suggested (Yasunaga and Hayashi 2002). In 2004 another species of *Isometopidea* was described, *Isometopidea formosana* Lin, and the next year it was transferred to the genus *Astroscopometopus* (Lin 2005). The same year Yasunaga (2005) described a new species, representing a new genus, *Kohnometopus fraxini* Yasunaga, 2005 in the tribe Myiommini Bergroth, 1924. Next, Akingbohungbe (2012) described the second representative of the genus *Gigantometopus*, *G. schuhi* Akingbohungbe, 2012. Yasunaga et al. (2017) transferred *Isometopidea yangi* (Lin 2005) to the genus *Kohnometopus*, suggested that this genus seemed better placed in Gigantometopini rather than in Myiommini, and also proposed to place the genus *Isometopidea* Poppius, 1913 (with the single species *Isometopidea lieweni* Poppius, 1913) in the tribe Gigantometopini. Moreover, it was found that the identity of the specimen of *I. lieweni* from Taiwan (Lin and Yang 2004) was based on a misidentification and it is a representative of an undescribed species. Subsequently Herczek et al. (2018) described one more genus and species within Gigantometopini, *Sulawesimetopus henryi* Herczek, Gorczyca & Taszakowski.

The most characteristic feature of Gigantometopini distinguishing it from other tribes is the large numbers of trichobothria (five or six on both mesofemur and metafemur) (Yasunaga et al. 2017).

In this paper, two new genera and species *Planicapitus luteus* gen. et sp. nov. and *Bruneimetopus simulans* gen. et sp. nov. are diagnosed and described; photographic images of habitus and genital structures, as well as scanning electron micrographs of the selected structures of both species are provided.

## Materials and methods

The specimens were imaged by the following equipment: Leica M205C stereo microscope with high diffuse dome illumination Leica LED5000 HDI, Leica DFC495 digital camera and Leica application suite 4.9.0 software; Leica DM 3000 upright light microscope with Leica MC 190 HD digital camera and Leica Application Suite 4.12.0 software. SEM photographs were obtained using Phenom XL field emission scanning electron microscope at 5 and 10 kV accelerating voltage with a BackScatter Detector (BSD). Graphic editor Adobe Photoshop CS6 was used to prepare the figures. In case of legs, the preparations for SEM were made with methods traditionally used in morphological studies (e.g. Kanturski et al. 2015, Herczek et al. 2018). In contrast, during preparation of other photographs, steps that can damage the specimen e.g., washing, dehydration and sputter-coating with a film of electrically conducting material, have not been applied. Specimens on original glue boards were only cleaned with a brush and mounted on aluminium stubs with double-sided adhesive carbon tape. Next, the specimens were covered with anti-static spray.

Map was prepared in SAGA GIS 7.1.1 (http://www.saga-gis.org) using WGS84 datum and EPSG: 3395 (World Mercator cylindrical projection).

Measurements were made with Leica application suite 4.9.0 software and are presented in millimetres (mm). Terminology of morphological structures mainly follows Herczek et al. (2018) and Kim and Jung (2019). Dissections of male genitalia were performed using Kerzhner and Konstantinov’s (1999) technique. The terminology for genital structures follows Konstantinov (2003). The study was based on material deposited in the collection of the Royal Belgian Institute of Natural Sciences (**RBINS**) and material recently collected by Claas Damken during an extensive survey of the Heteroptera fauna of Brunei Darussalam, deposited at Universiti Brunei Darussalam Museum, Brunei Darussalam (**UBDM**). From 2013 to 2015, sampling took place at different locations and forest types across the Bornean Sultanate using a range of methods (e.g., generator-powered light traps, sweep netting, collecting by hand, litter sifting, pitfall traps, Malaise traps, examination of bycatch from other studies). During this field survey, more than 400 species of Heteroptera were collected, including several species of Isometopinae (https://tinyurl.com/Brunei-Isometopinae).

## Taxonomy

### Planicapitus

Taxon classificationAnimaliaHemipteraMiridae

Taszakowski, Kim & Herczek gen. nov.

B76FB666-F888-5ABB-825A-625910625E6D

http://zoobank.org/E38884A2-CDD1-4C4F-95A5-E8AA1F979AA5

#### Type species.

*Planicapitus luteus* Taszakowski, Kim & Herczek, sp. nov.

#### Diagnosis.

Distinguished by vertical, flattened head, not punctured but wrinkled and distinctly higher than wide, dorsally extending to level of highest point of pronotum; vertex convex, protruding above eye level; width of vertex slightly larger than eye width; dorsum and pleurites of thorax with deep and dense punctures; calli slightly marked, tarsi two segmented, claw without subapical tooth; labium reaching third abdominal segment; right paramere very small, short, dagger-shaped; left paramere ca. 2.5 times as long as right one.

#### Description.

**Figure 1.**
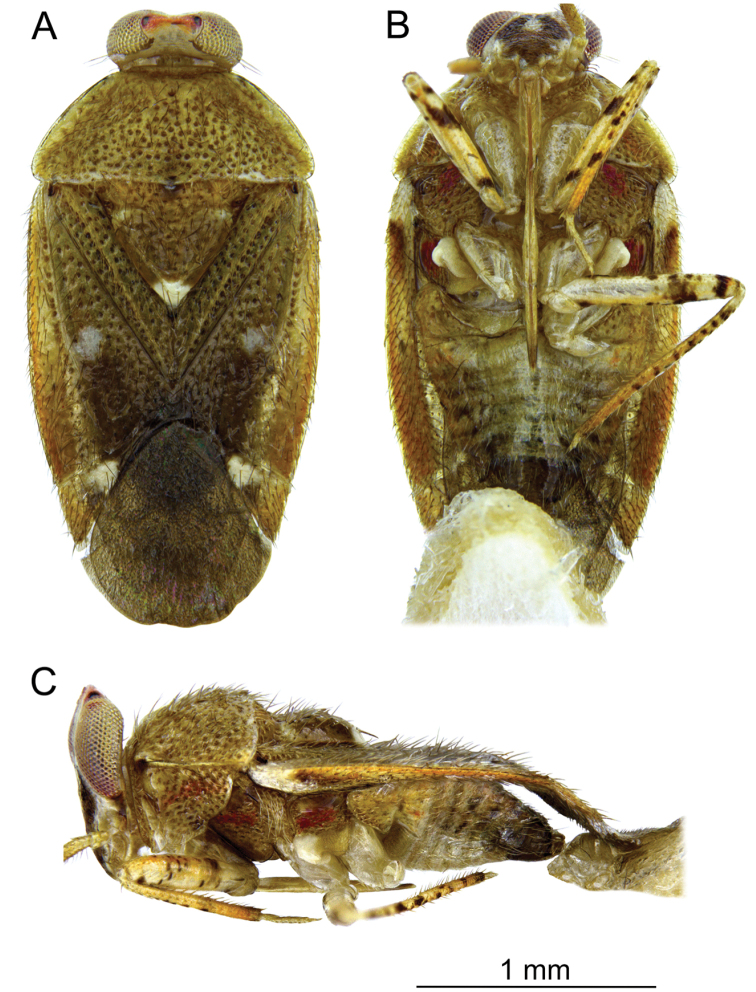
*Planicapitus luteus*: dorsal (**A**), ventral (**B**) and lateral (**C**) views.

**Male.** Body oval, slightly elongated (Fig. 1A). Head clearly higher than wide, dorsally extending to highest point of pronotum, flattened, impunctate but wrinkled; Antenna thin (particularly segments III and IV). Labium reaching third abdominal segment (Fig. 1B). Pronotal collar with row of punctures. Pronotum distinctly punctuate, distinctly carinate at sides, with slightly upturned lateral margins; calli slightly marked, separated by shallow fossa. Scutellum convex, wider than long, baso-medially clearly depressed. Thoracic pleura distinctly punctate (Fig. 1C). Ostiolar peritreme small, strongly convex and covered with fine spines (Fig. 4A–C). Mesofemora with five trichobothria (Fig. 3C, D). Tarsi two segmented, claws without apical tooth (Fig. 3 E–G). Genitalia: genital capsule trapeziform, with two longitudinal sutures at sides (Fig. 5A, B); aedeagus delicate, membranous, with weakly sclerotized dorsal wall of phallotheca, endosoma sacciform and membranous, weakly sclerotized (Fig. 5A, B, E). Left paramere scythe-shaped, sensory lobe with several long setae, apical process elongate (Fig. 5A–C); right paramere very small, short, dagger-shaped (Fig. 5A, B, D).

#### Remarks.

Affiliation of *Planicapitus luteus* to the Gigantometopini is clearly confirmed by the following features: compound eyes relatively small, significantly separated from each other, pronotum deeply punctate and elongate, calli separated by shallow fossa, pronotal collar demarcated by row of punctures, inflated scutellum, and five mesofemoral trichobothria (Herczek 1993, Yasunaga et al. 2017).

Set of features mentioned in the diagnosis clearly differ the new genus from other genera belonging to Gigantometopini. *Planicapitus luteus* belongs to the smallest representatives of tribe. The new genus is similar in size to *Isometopidea lieweni* which body length of the only known specimen equals to 3.0 mm. It is a female, so probably (like other representatives of tribe) males reach a smaller body size (Poppius 1913, Yasunaga 2005, Herczek et al. 2018). *Isometopidea* further differs from newly described genus by the structure of the head, which is not higher than wide, somewhat rounded and not strongly flattened in front. *Sulawesimetopus*, the second comparatively small-sized genus of the Gigantometopini, is slightly larger and can be distinguished from the new genus by the three segmented tarsi and punctured head. Other representatives of Gigantometopini are a way larger than the new genus in body size.

#### Etymology.

Combined from Latin adjective: *planus*, flat and noun: *caput*, *capitis*, head; gender masculine.

### Planicapitus luteus

Taxon classificationAnimaliaHemipteraMiridae

Taszakowski, Kim & Herczek sp. nov.

CD04D1B0-FC60-5407-B723-E88E7F396C7F

http://zoobank.org/F66DC352-A83C-4661-897A-D101551CDC2C

**Figure 2.**
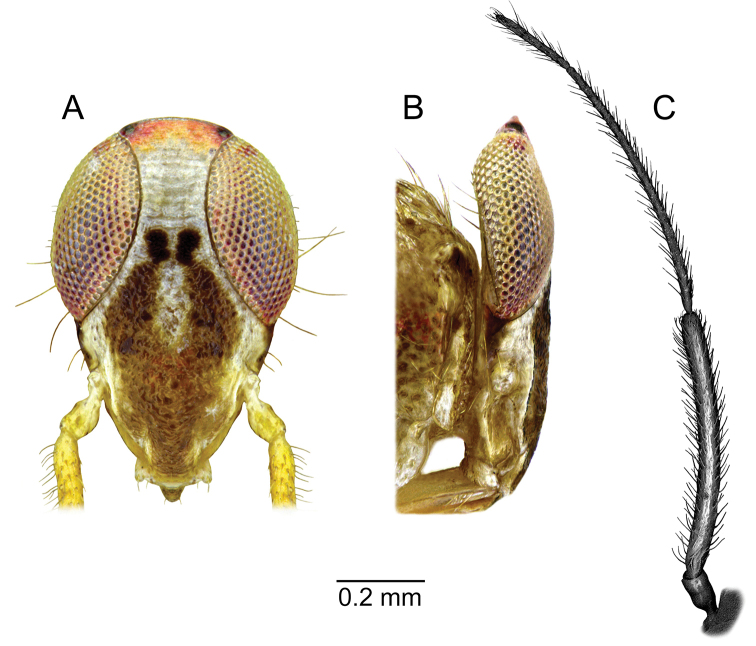
*Planicapitus luteus*: head in frontal view (**A**), lateral view of head (**B**), left antenna (**C**).

**Figure 3.**
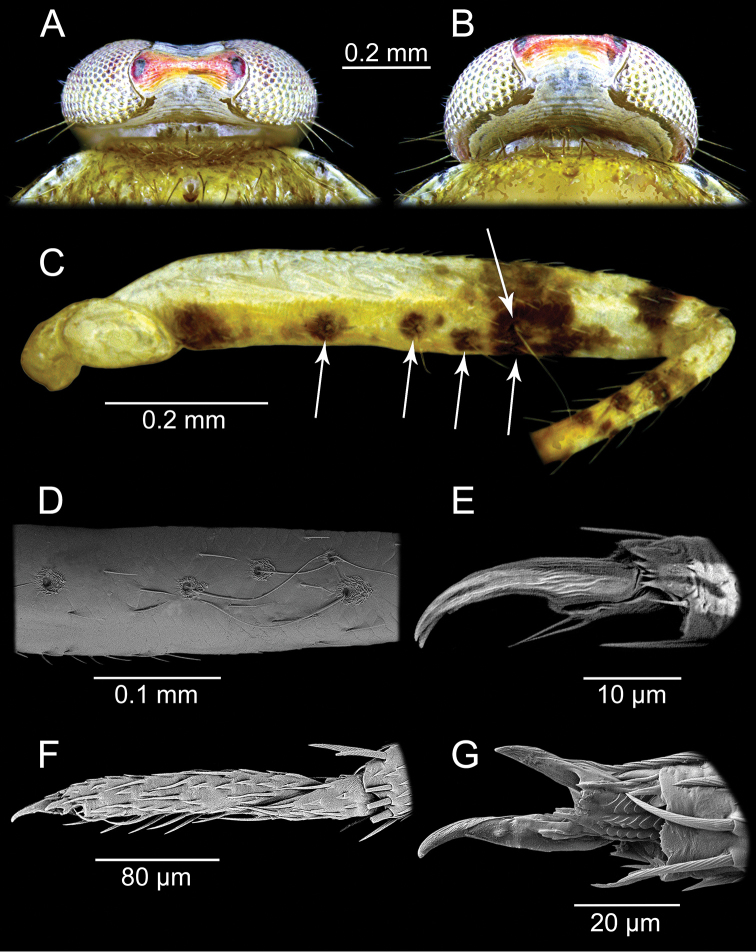
*Planicapitus luteus*: head in dorsal view (**A, B**), femur of middle leg in ventrolateral view, showing trichobothrial pattern (**C, D**), pretarsus of foreleg, lateral view (**E**), tarsus of middle leg, lateral view (**F**), pretarsus of middle leg, ventral view (**G**).

**Figure 4.**
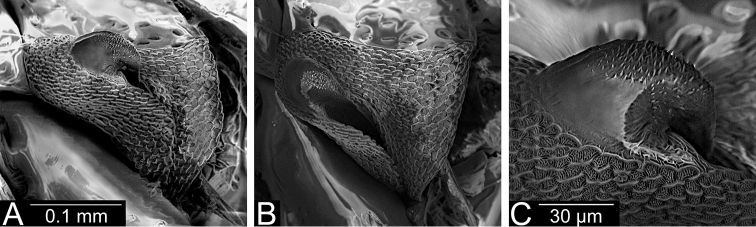
Scent gland, evaporatory area (**A, B**) and peritreme (**C**) of *Planicapitus luteus*.

**Figure 5.**
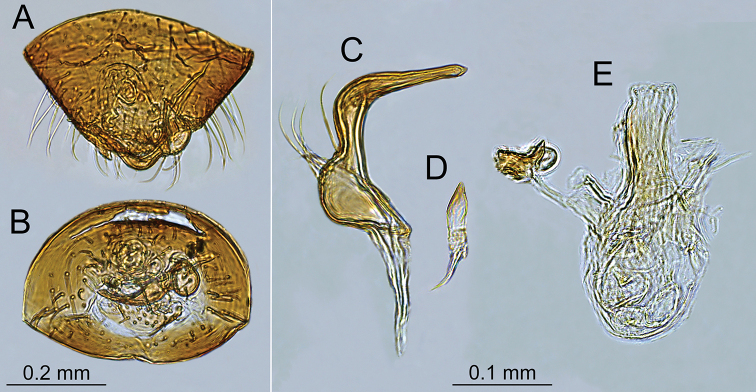
Male genitalia of *Planicapitus luteus*: genital capsule in dorsal (**A**) and caudal (**B**) views, left paramere (**C**), right paramere (**D**), aedeagus in dorsal view (**E**).

Figures 1 , 2 , 3 , 4 , 5

#### Diagnosis.

See generic diagnosis.

#### Description.

**Male.** Body shiny, yellow-brownish, covered by semi-erect pale-brown seta (Fig. 1A–C). ***Head***: yellow-whitish, 1.5 times as high as wide, compound eyes reddish yellow, vertex orange, convex, 1.3 times as wide as eye width in dorsal view. Frons whitish, with two small dark brown spots ventrally extending into large Y-shaped brown macula; gena whitish yellow (Figs 1C, 2A, B). Antenna yellowish. Labium shiny, yellowish, segment IV with brown apex (Fig. 1B). ***Thorax***: pronotum yellow, semi-transparent laterally; exposed part of mesoscutum yellow, scutellum yellowish brown, with apex white and lateral angles narrowly whitish, 0.7 as long as wide. Pleura yellowish brown, with red stripe from propleuron to episternum (Fig. 1B, C). Ostiolar peritreme ivory, evaporative area yellow (Fig. 1B, C). Claval commissure 0.6 times as long as scutellum. ***Hemelytron***: in various shades of yellow, median part with two whitish spots. Cuneus 0.9 times as long as wide, yellowish, with white spot in basal inner corner. Membrane pale grey, semi-transparent, with two cells. ***Legs***: coxae pale, almost white, femora yellow-white (Fig. 1B, C), with brown spots, tibiae yellow with dark brown spots, tarsi yellow (Fig. 1B, C). ***Abdomen***: bicoloured, dark brown, except for pale yellow genital segment (Fig. 1B, C). ***Genitalia***: as described above. ***Measurements***: given in the Table 1.

**Table 1.** Comparison of metric features of *Planicapitus luteus* and *Bruneimetopus simulans*.

	*P. luteus*	*B. simulans* holotype	*B. simulans* paratype	*B. simulans* average
Body length	2.61	2.52	2.47	2.50
Body width	1.24	1.13	–	1.13
Head length	0.22	0.19	0.19	0.19
Head width	0.58	0.51	0.52	0.52
Head height	0.86	0.71	0.68	0.71
Dorsal width of eye	0,20	0.17	0.19	0.18
Vertex width	0.26	0.17	0.19	0.18
Antennal segments I:II:III:IV	0.10:0.62:0.67:0.21	0.10:0.59:0.78:0.20	0.09:0.60:0.82:0.20	0.10:0.60:0.80:0.20
Labium length	1.30	1.26	–	1.26
Labial segments I:II:III:IV	0.35:0.26:0.30:0.37	0.34:0.36:0.23:0.39	–	0.34:0.36:0.23:0.39
Pronotum length	0.52	0.48	0.43	0.46
Anterior width of pronotum	0.51	0.48	0.44	0.46
Posterior width of pronotum	1.19	1.07	1.07	1.07
Mesoscutum length	0.10	0.12	0.10	0.11
Scutellum length	0.42	0.42	0.49	0.46
Scutellum width	0.61	0.68	0.59	0.64
Claval commissure	0.27	0.23	–	0.23
1^st^ femur length	0.73	0.64	0.68	0.66
1^st^ tibia length	0.76	0.73	0.76	0.75
1^st^ length of tarsus	0.26	0.20		
1^st^ length of tarsus I: II	0.09:0.22	0.08:0.17	0.08:0.17	0.08:0.17
2^nd^ femur length	0.86	0.73	0.73	0.73
2^nd^ tibia length	0.96	0,84	0.88	0.86
2^nd^ length of tarsus	0.21	0,19	0.19	0.19
2^nd^ length of tarsus I: II	0.08:0.18	0.08:0.16	0.07:0.15	0.08:0.16
3^rd^ femur length	–	–	0.92	0.92
3^rd^ femur width	–	–	0.23	0.23
3^rd^ tibia length	–	–	1.28	1.28
3^rd^ tarsus length	–	–	0.22	0.22
3^rd^ length of tarsus I: II	–	–	0.09:0.15	0.09:0.15
Heme length	1.99	1.84	–	1.84
Corium length	1.55	1.25	–	1.25
Cuneus length	0.29	0.23	–	0.23
Cuneus width	0.33	0.27	–	0.27

#### Etymology.

From Latin adjective *luteus*, yellow.

#### Biology.

Unknown.

#### Material examined.

***Holotype*** (♂): ‘Borneo, Sabah / Danum Valley / 70km W Lahad Datu / M.J. & J.P. Duffels // East Ridge Trail / 150m / 2.XII.1989 // sample Sab. 53 / understorey rainforest, / at light’. The holotype is deposited in RBINS.

### Bruneimetopus

Taxon classificationAnimaliaHemipteraMiridae

Taszakowski, Kim & Herczek gen. nov.

BE7C0E71-A1C2-5B70-96F6-DA7D2CD1A575

http://zoobank.org/63C3A9E7-12A1-4CB7-AB92-6F702663401D

#### Type species.

*Bruneimetopus simulans* Taszakowski, Kim & Herczek, sp. nov.

#### Diagnosis.

Distinguished by vertical, slightly flattened head, not punctured but wrinkled and higher than wide, dorsally not extending to level of highest point of pronotum; vertex slightly convex, protruding above eye level, width of vertex equal to eye width; dorsum and pleurites of thorax with deep and dense punctures; calli slightly marked, tarsi two segmented, claw with very small, barely noticeable apical tooth; labium reaching third abdominal segment, right paramere well developed, with knee-shaped sensory lobe; left paramere ca. 1.5 times as long as right one.

#### Description.

**Figure 6.**
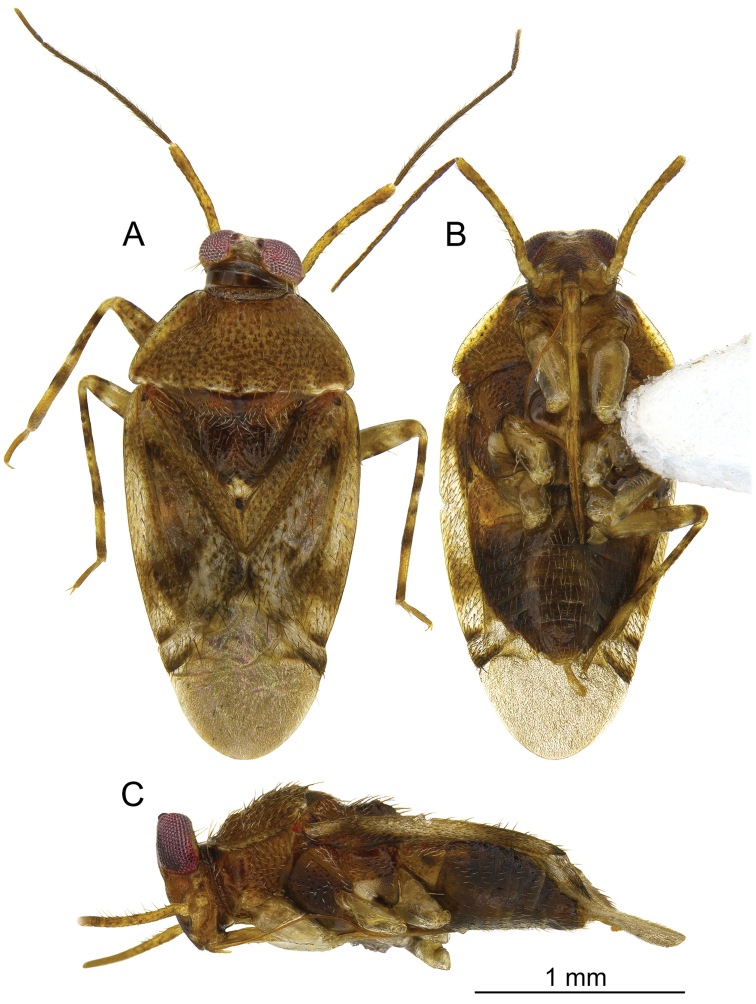
*Bruneimetopus simulans*: holotype: dorsal (**A**), ventral (**B**) and lateral (**C**) views.

**Figure 7.**
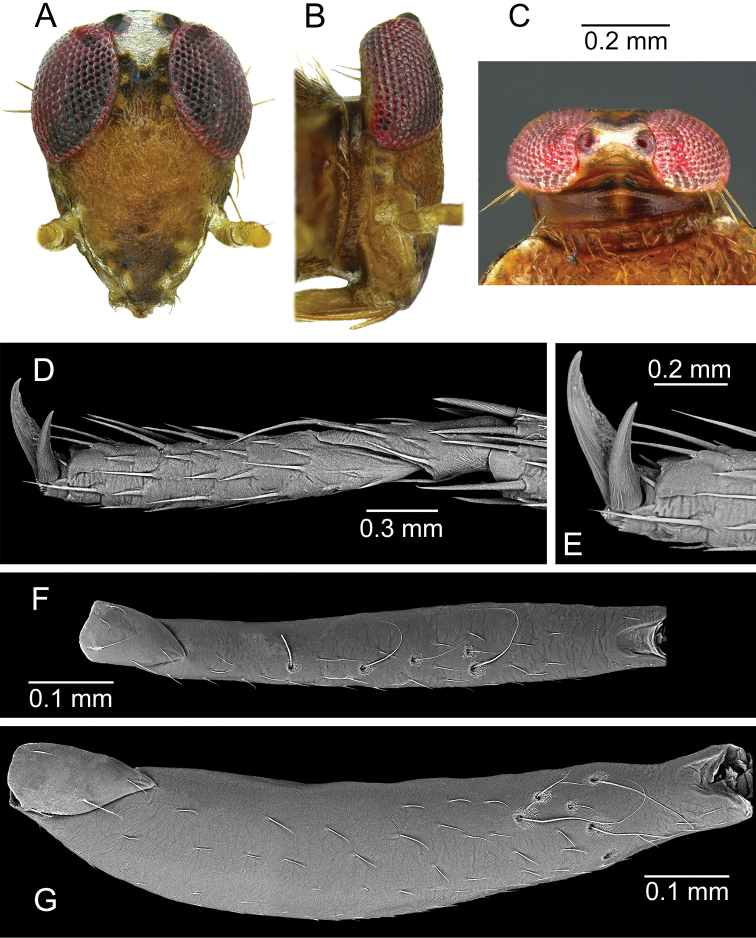
*Bruneimetopus simulans*: holotype: head in frontal view (**A**), lateral view of head (**B**), head in dorsal view (**C**), tarsus of middle leg, lateral view (**D**), pretarsus of middle leg, lateral view (**E**), paratype: femur of middle leg in ventral view, showing trichobothrial pattern (**F**), femur of hind leg in ventral view, showing trichobothrial pattern (**G**).

**Figure 8.**
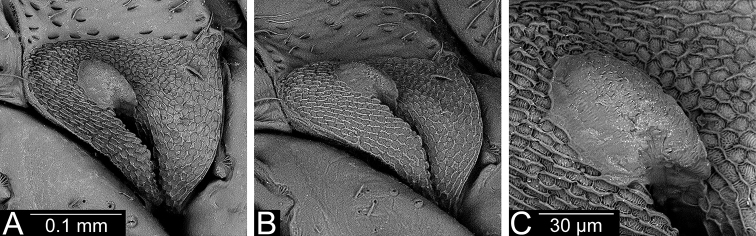
*Bruneimetopus simulans* (holotype) scent gland, evaporatory area (**A, B**) and peritreme (**C**).

**Figure 9.**
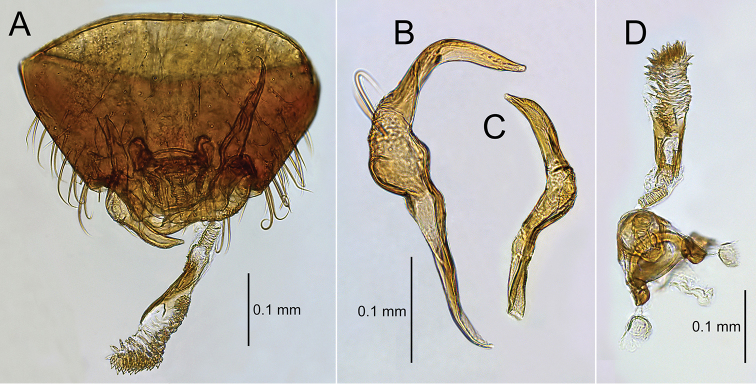
Male genitalia of *Bruneimetopus simulans*: holotype: genital capsule in dorsal view (**A**), left paramere (**B**), right paramere (**C**), aedeagus (**D**).

**Male.** Body oval, slightly elongate (Fig. 6A). Head higher than wide, dorsally not extending to highest point of pronotum, slightly flattened, impunctate but wrinkled. Antenna thin (particularly segments III and IV). Labium reaching third abdominal segment. Pronotal collar with row of punctures posteriorly. Pronotum distinctly punctuate, calli slightly marked, separated by shallow fossa. Scutellum slightly convex, baso-medially clearly depressed. Thoracic pleura distinctly punctate. Ostiolar peritreme small, moderately convex and covered with very fine spines (Fig. 8). Mesofemora with five, metafemora with six trichobothria (Fig. 7F, G). Tarsi two segmented, claws with very small, barely noticeable apical tooth (Fig. 7D, E). Genitalia: genital capsule trapeziform (Fig. 9A), aedeagus delicate, endosoma sacciform and membranous, very weakly sclerotized inside, outer subapical and apical part more sclerotic, clothed with dense spinules (Fig. 9A, D). Left paramere scythe-shaped, sensory lobe with several long setae, apical process elongated (Fig. 9A, B); right paramere left paramere ca. 1.5 times as long as right one, with knee-shaped sensory lobe (Fig. 9C).

#### Remarks.

Affiliation of *Bruneimetopus* to the Gigantometopini is clearly confirmed by the same set of features as for *Planicapitus* (see above). It is also indicated by presence of six metafemoral trichobothria (the specimen of *Planicapitus luteus* is devoid of hindlegs).

As in the case of *Planicapitus*, set of features mentioned in the diagnosis clearly differ the new genus from other genera belonging to Gigantometopini. The newly described genera are very similar morphologically to each other. However, in addition to small differences in the proportions of body parts and coloration, they can easily be distinguished by the completely different shape and size of the right paramere. This was a premise to describe them in separate genera.

#### Etymology.

Name combines Brunei (the type locality) with part of the generic name *Isometopus*, the type genus of the subfamily.

### Bruneimetopus simulans

Taxon classificationAnimaliaHemipteraMiridae

Taszakowski, Kim & Herczek sp. nov.

2A4E5F52-9E0D-516F-9BD8-836DDFF22EBF

http://zoobank.org/40B3C1AB-7913-4AA4-9AE1-82B098AE5B23

Figures 6 , 7 , 8 , 9

#### Diagnosis.

See generic diagnosis.

#### Description.

**Male.** Body shiny, in various shades of yellow and brown, covered by semi-erect pale brown and brown setae (Fig. 6A–C). ***Head***: brownish yellow, 1.4 times as high as wide, compound eyes reddish, vertex white, slightly convex, as wide as eye in dorsal view. Frons dark brown between eyes, yellowish below inferior margin of eyes; clypeus brown; gena yellow (Fig. 7A–C). Antenna yellowish, segments III and IV darker. Labium shiny, yellowish, segment IV brown (Fig. 6B). ***Thorax***: pronotum dark yellow, lateral margins semi-transparent and slightly raised, slightly wider at front; posterior margin whitish. Exposed part of mesoscutum brown with yellow tinge. Scutellum dark brown, with white apical part and black extreme apex, 0.6 times as long as wide, covered by semi-erect setae. Propleuron dark yellow, meso- and metapleurons dark brown with dark yellow tinge. Ostiolar peritreme ivory, evaporative area yellow-brown (Fig. 4D–F). Claval commissure comparatively long, 0.5 times as long as scutellum. ***Hemelytron***: in various yellow and brown shades: median, posterior part and cuneus in 2/3 of their length semi-transparent, whitish yellow, base of hemelytra and clavus yellow-brown, part neighbouring with cuneal fracture and 1/3 length of cuneus dark brown. Cuneus 0.9 as long as wide. Membrane pale grey, semi-transparent, with two cells. ***Legs***: coxae yellowish pale, femora yellow-white, with brown spots apically, tibiae yellow with four or five dark brown, irregular rings, tarsi yellow (Fig. 6A, B). ***Abdomen***: bicolored: first two segments yellowish to brown, others dark brown (Fig. 6B, C). ***Genitalia***: as described above. ***Measurements***: given in the Table 1.

#### Etymology.

The species name *simulans* (resembling) is the present participle of the Latin verb *simulo* (to make like or to assume the appearance of anything), in allusion to the resemblance of this species to *Planicapitus luteus*.

#### Biology.

**Figure 10.**
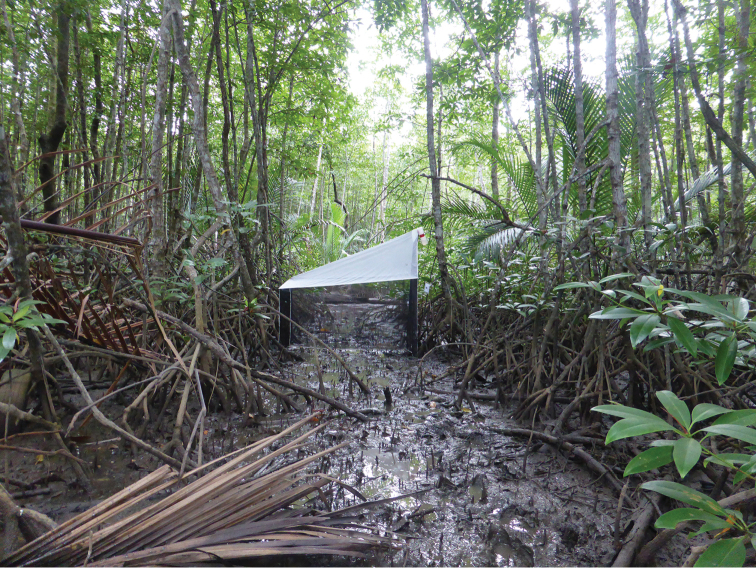
Malaise trap at the collecting site of holotype of *Bruneimetopus simulans*.

Unknown. Two specimens were collected in a mangrove forest (Fig. 10) by a Malaise trap, together with several other specimens of Isometopinae.

#### Material examined.

***Holotype*** (♂): ‘Borneo, Brunei, Tutong // Tutong area, mangroves forest / small stream near water edge, Malaise / trap 1; 16.viii.2014, leg: C. Damken / 4°46'9.54"N, 114°36'20.64"E // ID code: tutong.mangroves.01780’; ***Paratype*** (♂):‘Borneo, Brunei, Labu FR. / mangrove forest, Malaise trap ID4 / 06.viii.2018; leg: C. Damken / 4°50'53.11"N, 117°7'45.65"E// ID code: labu.mangroves.01777’. The holotype and paratype are deposited in the UBDM.

##### Distributional remarks

**Figure 11.**
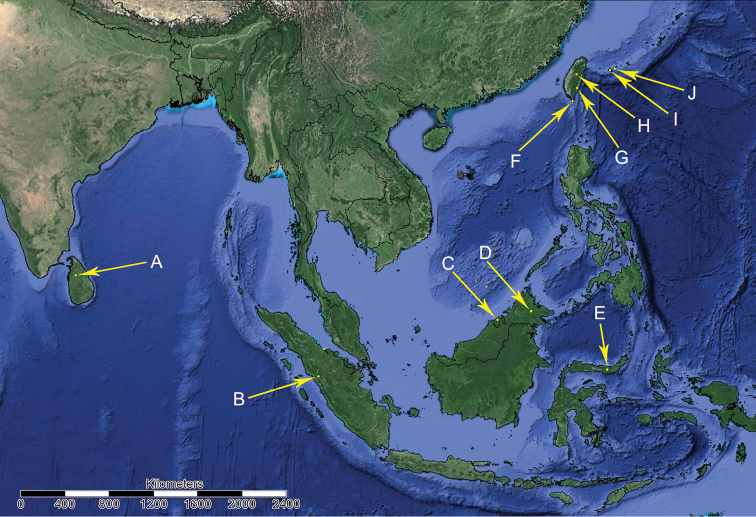
Distribution of Gigantometopini: **A***I. lieweni***B***G. rossi***C***G. schuhi* and *B. simulans***D***P. luteus***E***S. henryi***F***A. formosanus***G***K. yangi***H***A. formosanus* and *I. lieweni* nec **I***A. gryllocephalus***J***A. gryllocephalus* and *K. fraxini*

In total, only 49 imagines of Gigantometopini representing eleven species were ever recorded. Four species are known only from the holotype: *Gigantometopus rossi*, *Gigantometopus schuhi*, *Isometopidea lieweni*, and *Planicapitus luteus*. Below we present the complete checklist of Gigantometopini with distributional records (Fig. 11) and biological information (following Poppius 1913, Schwartz and Schuh 1990, Miyamoto et al. 1996, Yasunaga and Hayashi 1996, Lin and Yang 2004, Yasunaga 2005, Akingbohungbe 2012, Yasunaga et al. 2017, Herczek et al. 2018):

Gigantometopini Herczek, 1993

*Astroscopometopus* Yasunaga & Hayashi, 2002

***Astroscopometopus formosanus* (Lin, 2004)**

*Isometopidea formosana* Lin, 2004

1♂, Taiwan, Nantou, Chunyang (Fig. 11H), 11 Jun–9 Jul 2002, malaise trap

1♂, Taiwan, Pingtung, Hengchun, Kenting National Park (Fig. 11F), 10 Mar–14 Apr 2005, malaise trap

***Astroscopometopus gryllocephalus* (Miyamoto, Yasunaga, & Hayashi, 1996)**

*Isometopidea gryllocephala* Miyamoto, Yasunaga, & Hayashi, 1996

1♀, Japan, Ryukyu Arc., Ishigaki Is., Shiramizu (Fig. 11J), 19 Mar 1993, sweeping, grasses growing on the subtropical jungle floor near a montane stream.

1♀, Japan, Ryukyu Arc., Ishigaki Is., Mt. Yarabudake (Fig. 11J), 10 Mar 1999, the bark of the subtropical ash, *Fraxinus griffithii*

1♂, 8 final instar nymphs, Japan, Ryukyu Arc., Ishigaki Is., Mt. Fukami-Omoto (Fig. 11J), 18 Mar 2000, the bark of the subtropical ash, *Fraxinus griffithii*

1♀, Japan, Ryukyu Arc., Iriomote Is. (Fig. 11I), 2 Mar 2002, root of an unidentified broadleaved tree

*Gigantometopus* Schwartz & Schuh, 1990

***Gigantometopus rossi* Schwartz & Schuh, 1990**

1♀, Indonesia, Sumatra, Sumatera Barat, Mangani, mine near Kota Tinggi, 700 m a.s.l. (Fig. 11B), 20 Jul 1983

**Gigantometopus cf. rossi Schwartz & Schuh, 1990**

1♀, South India

***Gigantometopus schuhi* Akingbohungbe, 2012**

1♂, Brunei, Borneo, Bukit Sulang near Lamunin (Fig. 11C), 20 Aug–10 Sep 1982, insecticide fogging on *Shorea macrocarpa*

*Isometopidea* Poppius, 1913

***Isometopidea lieweni* Poppius, 1913**

1♀, Sri Lanka, Anuradhapura (Fig. 11A), 21 Dec

***Isometopidea lieweni* nec Poppius, 1913**

1♀, Taiwan, Nantou, Lienhachi (Fig. 11H), Nov 1984, malaise trap

*Kohnometopus* Yasunaga, 2005

***Kohnometopus fraxini* Yasunaga, 2005**

1♂, Japan, Ryukyu Arc., Ishigaki Is., Mt. Fukami-Omoto (Fig. 11J), 28 Sep 2002; 6♀♀, 22 May 2002

1♀, Japan, Ryukyu Arc., Ishigaki Is., Mt. Yarabudake (Fig. 11J), 1 Jun 2002; 2♂♂, 6♀♀, 28 Nov 2002; 1♀, 2 Oct 2002; all specimens of this species were collected on two trees of *Fraxinus griffithii*

***Kohnometopus yangi* (Lin, 2005)**

*Isometopidea yangi* Lin 2005

1♂, Taiwan, Taitung, Peinan Panchiu Station (Fig. 11G), 19 Nov–16 Dec 2004, malaise trap; 2♂♂, 2♀♀, 19 Nov–16 Dec 2004, malaise trap; 1♀, 7 Oct–19 Nov 2004, malaise trap; 3♂♂, 1♀, 16 Dec 2004–17 Feb 2005, malaise trap

*Sulawesimetopus* Herczek, Gorczyca & Taszakowski, 2018

***Sulawesimetopus henryi* Herczek, Gorczyca & Taszakowski, 2018**

3♂♂, Indonesia, Sulawesi Utara (Fig. 11E), 8–18 Nov 1985

5♂♂, 1♀, Indonesia, Sulawesi Utara, Dumonga-Bone National Park, Hogg’s Back Subcamp, 660 m. a.s.1. (Fig. 11E), 15 Nov 1985

*Planicapitus* Taszakowski, Kim & Herczek, gen. nov.

***Planicapitus luteus* Taszakowski, Kim & Herczek, sp. nov.**

1♂, Malaysia, Borneo, Sabah Danum Valley, East Ridge Trail, 150 m. a.s.1., (Fig. 11D), 2 Dec 1989, understorey rainforest, at light

*Bruneimetopus* Taszakowski, Kim & Herczek, gen. nov.

***Bruneimetopus simulans* Taszakowski, Kim & Herczek, sp. nov.**

1♂, Brunei, Borneo, Tutong area (Fig. 11C), 16 Aug 2014, mangroves forest (Fig. 10), malaise trap

1 ♂, Brunei, Borneo, Labu FR. (Fig. 11C), 6 Aug 2018, mangroves forest, malaise trap

## Supplementary Material

XML Treatment for Planicapitus

XML Treatment for Planicapitus luteus

XML Treatment for Bruneimetopus

XML Treatment for Bruneimetopus simulans
